# Optimized calorie and high protein intake *versus* recommended caloric-protein intake in critically ill patients: a prospective, randomized, controlled phase II clinical trial

**DOI:** 10.5935/0103-507X.20190025

**Published:** 2019

**Authors:** José Raimundo Araújo de Azevedo, Hugo Cesar Martins Lima, Widlani Sousa Montenegro, Suellen Christine de Carvalho Souza, Ivna Raquel Olimpio Moreira Nogueira, Marilia Martins Silva, Nicolli de Araujo Muniz

**Affiliations:** 1 Unidade de Terapia Intensiva, Hospital São Domingos - São Luís (MA), Brasil.

**Keywords:** Diet, high-protein, Energy intake, Food relief, Critical illness, Dieta rica em proteínas, Ingestão calórica, Auxílio nutricional, Estado crítico

## Abstract

**Objective:**

To evaluate differences in outcomes for an optimized calorie and high protein nutrition therapy versus standard nutrition care in critically ill adult patients.

**Methods:**

We randomized patients expected to stay in the intensive care unit for at least 3 days. In the optimized calorie and high protein nutrition group, caloric intake was determined by indirect calorimetry, and protein intake was established at 2.0 to 2.2g/kg/day. The control group received 25kcal/kg/day of calories and 1.4 to 1.5g/kg/day protein. The primary outcome was the physical component summary score obtained at 3 and 6 months. Secondary outcomes included handgrip strength at intensive care unit discharge, duration of mechanical ventilation and hospital mortality.

**Results:**

In total, 120 patients were included in the analysis. There was no significant difference between the two groups in calories received. However, the amount of protein received by the optimized calorie and high protein nutrition group was significantly higher compared with the control group. The physical component summary score at 3 and 6 months did not differ between the two groups nor did secondary outcomes. However, after adjusting for covariates, a negative delta protein (protein received minus predetermined protein requirement) was associated with a lower physical component summary score at 3 and 6 months postrandomization.

**Conclusion:**

In this study optimized calorie and high protein strategy did not appear to improve physical quality of life compared with standard nutrition care. However, after adjusting for covariates, a negative delta protein was associated with a lower physical component summary score at 3 and 6 months postrandomization. This association exists independently of the method of calculation of protein target.

## Abstract (pt)

**Objetivo:**

Avaliar as diferenças entre os desfechos da terapia nutricional com ingestão ideal de calorias mais alto teor proteico e do padrão de cuidados nutricionais em pacientes críticos adultos.

**Métodos:**

Randomizamos pacientes com previsão de permanecer na unidade de terapia intensiva por pelo menos 3 dias. No grupo com ingestão ideal de calorias mais alto teor proteico, a necessidade de ingestão calórica foi determinada por calorimetria indireta e a ingestão proteica foi estabelecida em níveis de 2,0 a 2,2g/kg/dia. O grupo controle recebeu calorias em nível de 25kcal/kg/dia e 1,4 a 1,5g/kg/dia de proteínas. O desfecho primário foi o escore do sumário do componente físico obtido aos 3 e 6 meses após a randomização. Os desfechos secundários incluíram força de preensão manual quando da alta da unidade de terapia intensiva, duração da ventilação mecânica e mortalidade hospitalar.

**Resultados:**

A análise incluiu 120 pacientes. Não houve diferença significante entre os dois grupos em termos de calorias recebidas. Contudo, a quantidade de proteínas recebidas pelo grupo com nível ideal de calorias mais alto teor de proteínas foi significantemente mais alta do que a recebida pelo grupo controle. O escore do sumário componente físico aos 3 e 6 meses após a randomização não diferiu entre ambos os grupos, assim como não diferiram os desfechos secundários. Entretanto, após ajuste para covariáveis, um delta proteico negativo (proteínas recebidas menos a necessidade proteica predeterminada) se associou com escore do sumário do componente físico mais baixo nas avaliações realizadas 3 e 6 meses após a randomização.

**Conclusão:**

Neste estudo, a estratégia com ingestão calórica ideal mais elevado teor proteico não pareceu melhorar a qualidade de vida física em comparação aos cuidados nutricionais padrão. Contudo, após ajuste para covariáveis, um delta proteico negativo se associou com escores do sumário do componente físico mais baixos nas avaliações realizadas aos 3 e aos 6 meses após a randomização. Esta associação ocorreu independentemente do método de cálculo do alvo proteico.

## INTRODUCTION

The importance of nutritional therapy for the critically ill patient has long been recognized. Critical illness is marked by an intense catabolic process associated with infectious and noninfectious complications and increased mortality.^(1)^ Severe disease survivors have significant muscle weakness and physical disability that may persist for years.^(2)^ Early initiation of nutritional therapy and optimal calorie and protein intake have a significant impact on outcomes in these patients.^(3)^ The Society of Critical Care Medicine (SCCM) and Society for Parenteral and Enteral Nutrition (A.S.P.E.N.) recently published Guidelines for the Provision and Assessment of Nutrition Support in the Adult Critically Ill Patient.^(4)^ The guidelines emphasize the importance of determining energy expenditure by indirect calorimetry as it is a more appropriate form to establish adequate caloric intake; however, the guidelines note that as this method is not available at most centers, caloric intake determination based on patient weight may be a viable alternative. In critically ill patients, protein is the most important macronutrient as it potentiates healing and immune function and helps patients maintain lean body mass.^(5)^ Most studies and guidelines recommend that critically ill patients receive 1.2 to 1.5 grams of protein per kilogram of body weight per day (g/kg/day). However, some observational studies suggest that a protein intake of 2.0 to 2.5g/kg/day could improve outcomes.^(6)^ Recent studies suggest that in critically ill patients, protein intake is more clearly related to outcome than the intake of other macronutrients and calories. In a prospective, observational study of patients in a medical/surgical intensive care unit (ICU), mortality decreased progressively as protein intake increased.^(7)^

At present, there is a clear need for an adequately powered, randomized study that analyses the impact of nutritional therapy on critically ill patients using patient-centered parameters as outcome measures. The study should compare high protein intake (2.0 to 2.2g/kg/day) with the recommended protein intake (1.2 to 1.5g/kg/day). The recently published EAT-ICU study^(8)^ compared critically ill patients undergoing nutritional therapy based on energy expenditure measured by indirect calorimetry and protein intake of at least 1.5g/kg/day with patients who had a goal of receiving 25kcal/kg/day calories and at least 1.2g/kg/day of protein. The primary outcome was the physical component summary (PCS) score, and no difference was found between the two groups when evaluating the PCS score at the end of 6 months. However, the study compared patients who received 97% of the caloric target and 1.47g/kg/day of protein with a control group that received only 64% of the caloric target and 0.5g/kg/day of protein. Therefore, in our view, the study does not answer the question of whether optimized caloric intake and high protein intake impact important outcomes in critically ill patients.

The aim of this study was to evaluate the effect of high protein intake of 2.0 to 2.2g/kg/day and caloric intake determined by indirect calorimetry versus recommended protein intake of 1.4 to 1.5g/kg/day and caloric intake of 25kcal/kg/day on outcomes in critically ill patients. The primary outcome to be investigated is the PCS of quality of life after 3 and 6 months of randomization, and additional secondary outcomes to be investigated include handgrip strength measured upon discharge from the ICU, duration of mechanical ventilation, ICU length of stay, and ICU and hospital mortality.

## METHODS

This is a prospective, randomized, controlled phase II trial conducted in both a surgical intensive care unit (13 beds) and a medical intensive care unit (32 beds) of a tertiary hospital. Included were patients over 18 years of age, nonpregnant, submitted to mechanical ventilation, expectation of stay in the ICU was greater than 2 days, and admitted to the ICU from June 2016 to November 2017. Patients were excluded if they were not expected to remain in the ICU for at least 3 days, had high-output bronchopleural fistulas, required an inspired fraction of oxygen (FiO_2_) ≥ 0.6, and presented evidence of severe cognitive dysfunction, which was identified through family information and patient evaluation performed by a hospital psychologist upon admission to the ICU. For patients who met the inclusion criteria, demographic data regarding age, gender, admissions category (medical or surgical), primary admission diagnosis, Acute Physiology and Chronic Health Evaluation (APACHE) IV score, admission SOFA and Nutrition Risk Score (NRS 2002) were collected. Written informed consent was obtained from the patient or a next of kin. The *Hospital São Domingos* Ethics in Research Committee approved the study (number 1.487.683).

Patients were randomized to the optimized calorie-high protein nutrition (OCHPN) group or the control group using a table of random numbers and sealed envelopes. After randomization into the two groups, nutrition therapy (preferably by the enteral route) was initiated as soon as possible and was allowed to progress over the following days to reach the caloric target. Those who could not achieve the caloric goal after 5 days of nutritional therapy received complementary parenteral nutrition. Patients who had high residue (greater than 300mL in 12 hours) within the first few hours of enteral nutritional therapy received metoclopramide IV and erythromycin enterally. If the high residue persisted on the third day of nutritional therapy, a postpyloric nutrition catheter was inserted. Patients with absolute contraindications to enteral nutrition received parenteral nutrition.

Patients in the study group had their resting energy expenditure measured daily by indirect calorimetry using GE-Carescape B650 (GE Healthcare Oy, Helsinki, Finland) equipment. In the first 3 days, the caloric intake was corrected daily to the value determined by indirect calorimetry. Then, until the 10th day of evolution, the caloric expenditure was corrected every 2 days. The protein intake of patients in the OCHPN group was set at 2.0 to 2.2g/kg/day. The nutritional formula used in this group was Peptamen Intense (1.0kcal/mL, 93 g/L protein, Nestle Health Care). Patients in the Control group had a caloric target of 25kcal/kg/day and a protein intake of 1.4 to 1.5g/kg/day. In this group, the formula used was preferably Novasource Senior (1.2kcal/ml, 65g/L protein, Nestle Health Care). Daily data on predicted and achieved caloric and protein intake was recorded for 14 days or until discharge or death.

The primary outcome was PCS score obtained from Medical Outcomes Study 36 - Item Short - Form Health Survey (SF-36) tool.^(9,10)^ The tool was validated for the Brazilian population, and responses were obtained by phone interview at 3 and 6 months after randomization. Secondary outcomes included handgrip strength measured at ICU discharge (Saehan Hydraulic Hand Dynamometer, Saehan Corp, Korea), duration of mechanical ventilation, ICU length of stay, and ICU and hospital mortality.

### Statistical analysis

Statistical analyses were performed using Statistical Package for Social Science (SPSS) software, version 20.0 (SPSS, Inc. an IBM Company, Chicago, IL). Continuous variables were assessed for normality using the Kolmogorov-Smirnov test. Parametric variables were compared between groups and within each group using the Student's t-test; nonparametric variables were compared using the Mann-Whitney test. Categorical variables were compared using the chi-square test. The primary analyses were performed in the intention-to-treat population, which included all randomized patients minus 18 patients (9 patients from each study group) who were excluded postrandomization. Patients who died before 3 and 6 months were given the lowest possible PCS score (Zero). The analyses were performed with and without adjustment for age, APACHE IV score, initial SOFA, nutrition risk score, admission category (clinical/surgical), energy received, energy balance, protein received and ∆ protein (protein received minus predetermined protein requirement). Receiver Operating Characteristic (ROC) curve analysis was used to determine the cut-off points for turning the nutritional support continuous variables (energy received, energy balance, protein received and ∆ protein) into binary variables. The allocation of patients in the groups (control and study) was performed through simple random sampling. The level of significance to reject the null hypothesis was 5%; thus, a p-value of < 0.05 was considered statistically significant.

## RESULTS

**Figure 1:**
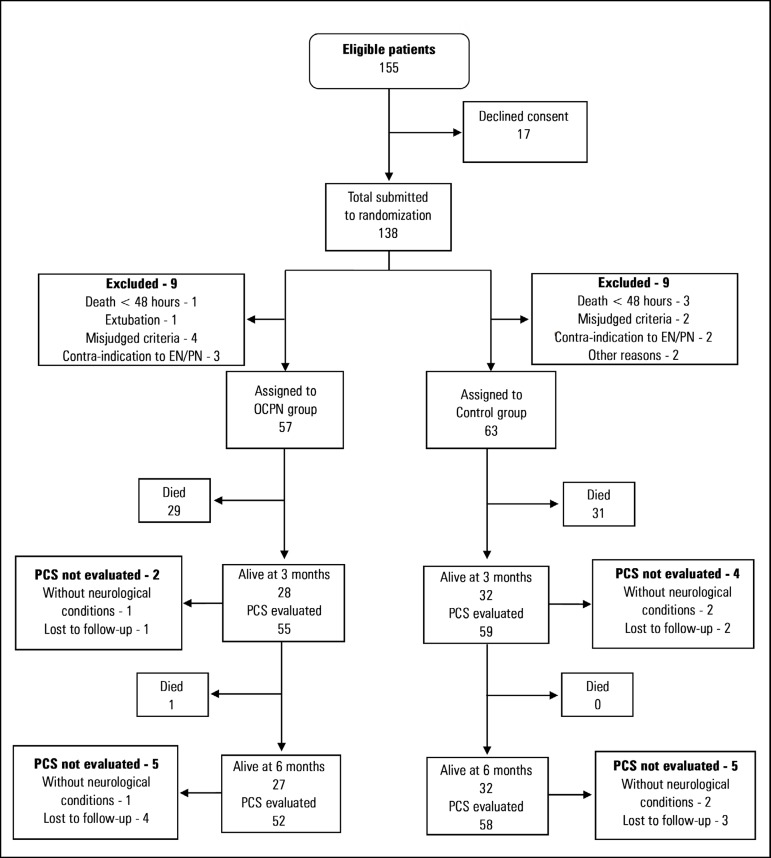
Flow diagram of study population. EN - enteral nutrition; PN - parenteral nutrition; OCPN - optimized caloric-protein nutrition; PCS - physical component summary.

Between June 2016 and November 2017, 155 patients fulfilled the inclusion criteria, and 17 declined the consent to participate. The remaining 138 patients were randomized into the OCHPN group (66) and the Control group (72). Eighteen patients with nine in each group were excluded postrandomization based on the reasons explained in figure 1. Thus, 120 patients were analyzed, including 57 in the OCHPN group and 63 in the Control group. Table 1 shows that demographic and clinical data were comparable between the two groups.

**Table 1:** Demographic and clinical characteristics of the patients

Variable	OCHPN group n = 57	Control group n = 63	p value
Age (years)	65.0 (18.8)	67.4 (18.9)	0.49
Female	23 (40.3)	31 (49.2)	0.33
APACHE IV score	81.1 (32.4)	77.2 (30.7)	0.50
SOFA baseline	9.8 (14.6)	6.8 (4.0)	0.11
Nutrition risk (NRS-2002)	3.9 (0.9)	4.1 (1.0)	0.22
Admission category			
Medical	46 (80)	46 (73)	
Surgical	11 (20)	17 (27)	
Primary ICU diagnosis			
Cardiovascular	17 (29.8)	23 (36.5)	
Respiratory	9 (15.7)	11 (17.4)	
Neurological	11 (19.2)	9 (14.2)	
Gastrointestinal	4 (7.0)	4 (6.3)	
Sepsis	12 (21.0)	15 (23.8)	
Other	4 (7.0)	1 (1.5)	

OCPN - Optimized Caloric-Protein Nutrition; APACHE IV - Acute Physiology and Chronic Health Evaluation IV; SOFA - Sequential Organ Failure Assessment; NRS-2002 - Nutrition Risk Score-2002. Results expressed as mean (standard deviation) or n (%).

### Nutrition therapy

The energy and protein pre-established requirements were significantly different between the two groups (Table 2). No significant differences were noted between the two groups in relation to the amount of calories received: 1139 calories (interquartile range [IR], 890.9 - 1278) in the OCHPN group and 1140 calories (IR, 889 - 1331) in the Control group (p = 0.70). On the other hand, the amount of protein received by the OCHPN group (1.69g/kg/day; IR, 1.33 - 1.80) was significantly higher than the amount received by the Control group (1.13g/kg/day; IQ, 0.97 - 1.34) (p < 0.0001). Optimized calorie-high protein nutrition patients received 73.2% of the energy expenditure determined by indirect calorimetry and 80% of the predetermined protein intake of 2.1g/kg/day. Patients in the Control group received 78% of the estimated energy expenditure of 25kcal/kg/day and 77.9% of the predetermined protein intake of 1.45g/kg/day.

**Table 2:** Nutrition therapy

Variable	OCHPN group n = 57	Control group n = 63	p value
Estimated/measured energy expenditure (kcal/day)	1554* (1383 - 1862)	1450† (1300 - 1625)	0.02
Predetermined protein requirement (g/kg/day)	2.1 (2.1 - 2.1)	1.45 (1.45 - 1.45)	< 0.0001
Nutrition received			
Total calories received (kcal/day)	1139 (890 - 1278)	1140 (889 - 1331)	0.70
Total protein received (g/kg/day)	1.69 (1.33 - 1.80)	1.13 (0.97 - 1.34)	< 0.0001
Energy balance‡ (kcal/day)	- 488 (-895 - -278)	- 353.7 (-549.5 - -122.5)	0.002
Delta protein§ (g/day)	-0.41 (-0.77 - -0.30)	-0.32 (- 0.48 - -0.11)	0.001

OCPN - Optimized Caloric-Protein Nutrition.

* Measured by indirect calorimetry;

† Calculated as 25 kcal/kg/day;

‡ Energy balance was calculated as total calories received minus measured energy expenditure per day;

§ Delta protein was calculated as protein received minus predetermined protein requirements. Values expressed as median (interquartile range).

### Primary outcomes: physical component summary scores after 3 and 6 months

The PCS score was assessed 3 months after randomization in 55 (96.4%) patients in the OCHPN group and 59 (93.6%) patients in the Control group. Of these, 29 (52.7%) patients in the OCHPN group and 31 (52.5%) patients in the Control group died and received a zero in the PCS score. Six months after randomization, the PCS score was assessed in 52 (91.2%) patients in the OCHPN group and 58 (92.0%) patients in the Control group. Of these, 30 (71.4%) patients in the OCHPN group and 31 (55.5%) patients in the Control group died and received zero in the PCS score. There was no significant difference between groups regarding PCS outcomes at 3 and 6 months after randomization. However, in the multivariate analysis, after adjusting for independent covariates including age, APACHE IV score, admission SOFA, NRS-2002, admission category, energy balance and ∆ protein (protein intake relative to goal), a negative ∆ protein was associated with a lower PCS score at 3 months (OR, 2.63; 95%CI, 1.02 - 6.76; p = 0.045) and at 6 months (OR, 3.26; 95%CI, 1.21 - 8.80; p = 0.019), while a negative caloric balance did not influence PCS score at 3 months (OR, 1.91; 95%CI, 0.63 - 5.78; p = 0.255) or 6 months (OR, 2.67; 95%CI, 0.86 - 8.24; p = 0.089) (Tables 3 and 4).

**Table 3:** Multivariate logistic regression analysis of 3-month physical component summary

Variables	Wald	p value	OR	95%CI for OR	
				Lower	Upper
Age	6.20	0.013	3.05	1.27	7.34
APACHE IV	0.66	0.417	1.53	0.55	4.27
SOFA score	0.26	0.607	0.78	0.30	2.03
Calories received (median)	3.52	0.061	0.33	0.10	1.05
Proteins received (median)	1.66	0.198	1.99	0.70	5.64
Cal balance	1.30	0.255	1.91	0.63	5.78
Delta protein	4.01	0.045	2.63	1.02	6.76

OR - odds ratio; 95%CI - 95% confidence interval; APACHE IV - Acute Physiology and Chronic Health Evaluation IV; SOFA - Sequential Organ Failure Assessment; Delta protein was calculated as protein received minus predetermined protein requirements.

**Table 4:** Multivariate logistic regression analysis of 6-month physical component summary

Variables	Wald	p value	OR	95%CI for OR	
				Lower	Upper
Age	4.25	0.039	2.53	1.05	6.13
APACHE IV	0.23	0.635	0.78	0.28	2.19
SOFA score	0.48	0.488	1.44	0.51	4.07
Calories received (median)	3.85	0.049	0.30	0.09	1.00
Proteins received (median)	1.36	0.244	2.19	0.59	8.15
Cal balance	2.90	0.089	2.67	0.86	8.24
Delta protein	5.46	0.019	3.26	1.21	8.80

OR - odds ratio; 95%CI - 95% confidence interval; APACHE IV - Acute Physiology and Chronic Health Evaluation IV; SOFA - Sequential Organ Failure Assessment; Delta protein was calculated as protein received minus predetermined protein requirements.

### Secondary outcomes

Handgrip strength was evaluated at the time of ICU discharge in 24 patients in the OCHPN group and 27 patients in the Control group (Table 5). There was no significant difference in handgrip strength for males in the OCHPN group (median, 18kgf; IQ, 15 - 25) *versus* the Control group (median, 23.5kg; IQ, 13.7 - 32.0) (p = 0.35). Similarly, there was no significant difference in handgrip strength for females in the OCHPN group (median, 8kgf; IQ, 2 - 17) *versus* the Control group (median, 14.0kgf; IQ, 7.0 - 22.5) (p = 0.18). The other secondary outcomes, including ICU LOS, duration of mechanical ventilation, and ICU and hospital mortality, did not present any significant differences between the two groups.

**Table 5:** Primary and secondary outcome measures

Variable	OCHPN group n = 57	Control group n = 63	p value
Primary outcome measures			
PCS score at 3 months	n = 55 93.6 (126.1)	n = 59 85.2 (110.6)	0.70
PCS score at 6 months	n = 52 92.0 (133.4)	n = 58 90.0 (120.6)	0.93
Secondary outcome measures			
Handgrip at ICU discharge (kgf)			
Male	n = 15 18 (15 - 25)	n = 14 23.5 (13.7 - 32.0)	0.35
Female	n = 9 8.0 (2 - 17)	n = 13 14 (7.0 - 22.5)	0.18
ICU LOS	21 (13 - 33)	18 (10 - 35)	0.56
Duration of mechanical ventilation	9 (5 - 14)	9 (5 - 14)	0.64
ICU mortality	22 (38.5)	28 (44.4)	0.69
Hospital mortality	26 (45.6)	29 (46.0)	0.88

OCPN - Optimized Caloric-Protein Nutrition; PCS - physical component summary; ICU - intensive care unit; LOS - length of stay. Values expressed as mean (standard deviation), median (interquartile range) or n (%).

## DISCUSSION

In this prospective, randomized, controlled phase II trial, we analyzed 120 critically ill adult patients who were subjected to mechanical ventilation. The two groups received similar caloric intake, but the OCHPN group received significantly higher protein intake compared with the Control group. After adjusting for preselected covariates, a negative ∆ protein, i.e., receiving less than the predicted protein target, was associated with a lower PCS score at 3 and 6 months postrandomization. On the other hand, a negative caloric balance did not influence the PCS score at 3 or 6 months postrandomization. There was no difference between the groups regarding the secondary outcomes represented by the handgrip strength measurement at ICU discharge, ICU length of stay, duration of mechanical ventilation, and ICU and hospital mortality.

In a recent study, Allingstrup et al.^(8)^ analyzed 199 patients randomized to receive caloric intake determined by indirect calorimetry and high protein intake compared with a group receiving 25kcal/kg/day and usual protein intake. The study found no difference in the PCS score for quality of life between the two groups when assessed 6 months after randomization. It should be emphasized that in our study, the OCHPN group received 73.2% of the energy expenditure determined and 80% of the preset protein intake, and the control group received 78% of the estimated energy expenditure and 77.9% of the predetermined protein intake. However, in the Alingstrup et al. trial, the study group received 97% of the energy expenditure determined and 97% of the pre-established protein intake, while the control group received only 64% of the caloric intake and 45% of the pre-established protein intake. This difference in % calorie/protein received could explain why our study found a different result regarding quality of life.

In our study, 22.5% of the patients had sepsis and septic shock. In the Alingstrup study, 47% of the patients had sepsis and septic shock. Some studies have shown that protein kinetics are different in septic and nonseptic patients.^(11,12)^ The benefits of high protein intake are clearly identified in nonseptic patients, but there is no evidence that there are benefits in septic patients. In any case, we understand that the disease distribution in our study was within the epidemiological profile of general ICUs.

Other studies compared different nutritional regimens by analyzing short and long-term outcomes. Ferrie et al.^(13)^ randomized ICU patients to receive parenteral nutrition with 1.2g/kg/day of protein compared with 0.8g/kg/day and did not observe significant differences in short-term outcomes. The main criticism of the study concerns the reduced protein intake used in both groups and the fact that at least half of the patients analyzed were elective, low severity, surgical patients. In a retrospective study, Wei et al.^(14)^ compared patients who received different caloric intake and analyzed mortality and quality of life after 3 and 6 months. Wei at al. demonstrated that after adjustment for covariates, the group that received full caloric intake exhibited reductions in mortality and 2 components (physical functioning and role physical) of SF-36 after 3 months but not after 6 months. Other studies^(15,16)^ also analyzed the impact of different caloric intake on mortality at 6 and 12 months. However, it should be emphasized that these studies did not consider the impact of protein intake in the analysis.

Prospective observational studies suggest that achieving the prescribed protein target during critical illness is more likely to improve ICU outcomes than meeting energy goals. In a cohort of 113 patients, Allingstrup et al.^(7)^ reported lower 28-day mortality per gram of protein intake; however, greater energy intake did not provide a significant benefit. In a cohort of 726 nonseptic ICU patients, Weijs et al.^(17)^ found that mortality was lower with greater protein intake but increased with energy overfeeding. Nicolo et al.^(18)^ analyzed 2824 critically ill patients who remained in the ICU for at least 4 days to evaluate the impact of protein delivery on mortality and observed that administration of greater than 80% of goal protein was associated with a 40% reduction in mortality. In contrast, an increase in energy delivery was not associated with a reduction in mortality. These results from observational studies have led clinicians to suggest that IV amino acids provided in the form of supplemental parenteral nutrition should be added to insufficient enteral nutrition to optimize outcome. However, the results from an RCT suggest that minimal added benefits result from such strategies.^(19-21)^

The major strength of this study is that it is a prospective randomized trial. Patients in the study group received caloric intake based on indirect calorimetry and high protein intake and were compared to patients who received 25kcal/kg/day of calories and 1.4 to 1.5g/kg/day of protein. Another strength is that although we included patients expected to stay in the ICU for at least 3 days, most patients included in the analysis remained in the ICU for at least 10 days. As a result, patients were submitted to mechanical ventilation for 5 or more days, which allowed us to avoid the confounding effect of a short-term stay in the ICU since these patients would receive short-term nutritional support and usually have a good outcome. Thus, we studied a group of truly critically ill patients.

Our study is not without limitations. This study included patients who were admitted to two ICUs from the same hospital. Additionally, recent concepts, such as autophagy, which is a physiologic mechanism to remove dysfunctional and toxic proteins that is inhibited by early protein provision, and the endogenous production of calories in the first days of critically ill patient management can result in overfeeding if we are not careful to use full caloric intake only after 5 to 7 days of evolution. In our study, nutrition therapy was initiated as soon as possible after admission, and the caloric and protein target was achieved until the fifth day of nutritional therapy.

The main weakness of both our study and the Allingstrup study was that we did not examine systematic physical activity represented by resistive exercise in association with nutrition therapy. Recently, the Journal of Intensive Care Medicine published a research agenda on nutrition and metabolism in critically ill patients.^(22)^ At the top of the agenda is the need for prospective studies on protein intake associated with physical activity. Several recent publications have addressed physical exercise as a method to improve the outcome of patients hospitalized in ICUs.^(23-25)^ However, studies that combine optimal nutritional intake with an exercise program are lacking. There are also missing definitions on when to start physical activity and which exercises to use.

## CONCLUSION

In this study, an optimized caloric and high protein strategy did not appear to improve physical quality of life or other important outcomes compared with the standard nutritional care program. However, after adjusting for important covariates, we found that receiving less than the predicted protein target was associated with a lower physical component summary score at 3 and 6 months regardless of whether the protein target was 2.0 - 2.2g/kg/day or 1.4 to 1.5g/kg/day.
